# High inter-rater reliability in consensus diagnoses and overall assessment in the Asian Cohort for Alzheimer’s Disease Study

**DOI:** 10.1038/s44400-025-00015-1

**Published:** 2025-07-30

**Authors:** Yara Alkhodair, Ging-Yuek R. Hsiung, Boon Lead Tee, Pei-Chuan Ho, Phoenix Au Yeung, Wai Haung Yu, Guerry M. Peavy, Victor W. Henderson, Yun-Beom Choi, Clara Li, Dolly Reyes-Dumeyer, Haeok Lee, Walter A. Kukull, The Clinical Core of the Asian Cohort for Alzheimer’s Disease (ACAD), Wai Haung Yu, Victor W. Henderson, Yun-Beom Choi, Clara Li, Dolly Reyes-Dumeyer, Haeok Lee, Walter A. Kukull, Howard H. Feldman, Yian Gu, Pei-Chuan Ho, Ging-Yuek Robin Hsiung, Lorene Leung, Collin Liu, Richard Mayeux, Guerry M. Peavy, Boon Lead Tee, Ellen C. Wong, Hyun-sik Yang, Jennifer S. Yokoyama, Helena C. Chui, Tiffany W. Chow, Gyungah R. Jun, Van M. Ta Park, Helena C. Chui, Li-San Wang, Tiffany W. Chow, The Asian Cohort for Alzheimer’s Disease Study, Gyungah R. Jun, Li-San Wang, Tatiana Foroud, Joshua D. Grill, Maureen Kirsch, Wan-Ping Lee, Mingyao Li, Van M. Ta Park, Gerard D. Schellenberg, Mina Torres, Marian Tzuang, Badri N. Vardarajan, Rohit Varma, Eugene Yau

**Affiliations:** 1 Division of Neurology, University of British Columbia, S151 – 2211 Wesbrook Mall, UBC Hospital, Vancouver, BC V6T 2B5 Canada; 2 Djavad Mowafaghian Centre of Brain Health, University of British Columbia, 2215 Wesbrook Mall, Vancouver, BC V6T 1Z3 Canada; 3 Neuroscience Centre of Excellence, King Faisal Specialist Hospital and Research Centre, Riyadh, Saudi Arabia; 4 Memory and Aging Center, Department of Neurology, Weill Institute for Neurosciences, University of California, San Francisco, 1651 4th St Suite 212, San Francisco, CA 94158 USA; 5 Global Brain Health Institute, University of California, San Francisco, 1651 4th St, 3rd Floor, San Francisco, CA 94143 USA; 6 Penn Neurodegeneration Genomics Center, Department of Pathology and Laboratory Medicine, Perelman School of Medicine, University of Pennsylvania, 3700 Hamilton Walk, Richards Building D101, Philadelphia, PA 19104 USA; 7 The Leonard and Davis Institute of Health Economics, University of Pennsylvania, 3641 Locust Walk, Philadelphia, PA 19104 USA; 8 Department of Pharmacology and Toxicology, University of Toronto, Medical Sciences Building, King’s College Cir Room 4207, Toronto, ON M5S 1A8 Canada; 9 Department of Neurosciences, University of California at San Diego, 9500 Gilman Dr, La Jolla, CA 92093 USA; 10 Department of Epidemiology and Population Health, Stanford University, 291 Campus Drive, Li Ka Shing Building, Stanford, CA 94305 USA; 11 Department of Neurology & Neurological Sciences, Stanford University, 291 Campus Drive, Li Ka Shing Building, Stanford, CA 94305 USA; 12 Englewood Health, 350 Engle Street, Englewood, NJ 07631 USA; 13 Department of Neurology, Rutgers New Jersey Medical School, 90 Bergen Street, Doctors Office Center, Suite 5200, Newark, NJ 07101 USA; 14 Alzheimer’s Disease Research Center, Department of Psychiatry, Icahn School of Medicine at Mount Sinai, 1 Gustave L. Levy Place, New York, NY 10029 USA; 15 Gertrude H. Sergievsky Center, Taub Institute of Aging Brain and Department of Neurology at Columbia University, 630 West 168th Street, New York, NY 10032 USA; 16 Rory Meyers College of Nursing, New York University, 433 1st Ave, New York, NY 10010 USA; 17 Department of Epidemiology, University of Washington, 3980 15th Ave NE, Seattle, WA 98195 USA; 18 Department of Medicine (Biomedical Genetics), Boston University School of Medicine, 72 East Concord Street E200, Boston, MA 02118 USA; 19 Department of Ophthalmology, Boston University School of Medicine, 85 E Concord St, Boston, MA 02118 USA; 20 Department of Biostatistics, Boston University School of Public Health, 715 Albany St, Boston, MA 02118 USA; 21 University of California, San Francisco School of Nursing, 2 Koret Way, San Francisco, CA 94143 USA; 22 Department of Neurology, Keck School of Medicine at University of Southern California, 1975 Zonal Ave, Los Angeles, CA 90033 USA; 23 Department of Neurology and the Taub Institute for Research on Alzheimer’s Disease and the Aging Brain, Columbia University, Vagelos College of Physicians and Surgeons, 710 West 168th Street, New York, NY 10032 USA; 24 Alzheimer’s Disease Cooperative Study, University of California, San Diego, 9500 Gilman Dr, MC0949 San Diego, USA; 25 Department of Neurology, Columbia University Medical Center, 710 West 168th Street, New York, NY 10032 USA; 26 Center for Alzheimer Research and Treatment, Department of Neurology, Brigham and Women’s Hospital, 60 Fenwood Road, Hale Building for Transformative Medicine, Boston, MA 02115 USA; 27 Harvard Medical School, 25 Shattuck St, Boston, MA 02115 USA; 28 Department of Radiology and Biomedical Imaging, University of California, San Francisco, 505 Parnassus Ave, San Francisco, CA 94143 USA; 29 Department of Medical and Molecular Genetics, Indiana University School of Medicine, 340 West 10th Street, Fairbanks Hall, Suite 6200, Indianapolis, IN 46202 USA; 30 University of California Irvine Institute for Memory Impairments and Neurological Disorders, University of California, Irvine, Irvine, CA USA; 31 Department of Biostatistics, Epidemiology and Informatics, Perelman School of Medicine, University of Pennsylvania, 423 Guardian Drive, Blockley Hall, Philadelphia, PA 19104 USA; 32 Southern California Eye Institute, CHA Hollywood Presbyterian Medical Center, 1300 North Vermont Avenue, Doctors Tower, Ste 101, Los Angeles, CA 90027 USA; 33 Department of Community Health Systems, University of California, San Francisco School of Nursing, 2 Koret Way, San Francisco, CA 94143 USA

**Keywords:** Alzheimer's disease, Dementia

## Abstract

The Asian Cohort for Alzheimer’s Disease (ACAD) study is a collaborative investigation of genetic and non-genetic risk factors for AD among Asian Americans and Canadians. Harmonization of diagnostic procedures across recruiting sites will be key to the dataset’s efficacy.

Forty-two participants who completed the consensus process across seven ACAD recruiting sites were re-reviewed by two further impartial raters. Cohen’s Kappa coefficient was used to evaluate inter-rater agreement. The findings reveal the highest level of observed agreement at 88% and a Cohen’s Kappa of 0.835, among site consensus participants and two levels of external review, affirming the reliability of our protocol. ACAD has developed a data collection and diagnostic process that allows consistency among sites that serve Asians speaking Korean, Chinese, and Vietnamese languages.

## Introduction

The Asian Cohort for Alzheimer’s Disease (ACAD) investigates genetic and non-genetic factors influencing Alzheimer’s disease (AD) risk in Asian American and Canadian populations, which remain underrepresented in AD research. The protocol includes clinical evaluations, cognitive testing, and questionnaires on early life experiences and lifestyle. Validated assessments are administered in English, Cantonese, Mandarin, Korean, and/or Vietnamese^1^.

Inter-rater reliability is essential in studies of neurodegenerative disorders like AD, ensuring consistent diagnoses that support research integrity and valid sample selection. Accurate diagnostic agreement enhances the quality of study outcomes and clinical interpretations. However, variability in clinical judgment can hinder reliability and complicate the interpretation of findings^2,3^.

ACAD’s protocol assesses and diagnoses participants across seven recruiting sites^1^. We conducted the current study to evaluate the consistency of ACAD’s diagnostic procedures across sites by comparing agreement levels among site consensus meeting results approved by reviewers external to that process and a second round of independent raters. By analyzing agreement rates and calculating Cohen’s Kappa, the study aimed to identify areas of disagreement and investigate the reasons behind these differences. This would help us improve the ACAD data collection protocol, with the intention to minimize biases and differences in judgments among clinical raters and to improve the trustworthiness of the results.

Each ACAD site conducted its own consensus diagnosis meetings attended by a quorum of two clinicians experienced in dementia and at least one rater who administered the data collection packet. A summary of the evidence to support the diagnosis of cognitively normal, mild cognitive impairment (MCI), or dementia is completed after the meeting for entry into the REDCap electronic database. An important step on this form is for sites to designate an Overall Assessment indicating whether there is a history of loss of independent function and/or evidence of cognitive impairment on the testing administered by ACAD staff. The overall Assessment should help any inter-rater reliability reviewer to anticipate the diagnosis made.

It is important to note that raters only perform assessments and do not make diagnostic determinations. All diagnoses are made during site consensus meetings by qualified clinicians, based on standardized diagnostic criteria derived from the National Alzheimer’s Coordinating Center (NACC). During the current review process, several diagnostic discrepancies identified areas where interpretation guidelines could be more precise. These insights were used to revise the instructional materials and retrain site staff to improve consistency.

The ACAD Clinical Core determines the data collection protocol. Two reviewers external to each site (V.H. and T.W.C.) check the summaries for clinical consistency, confirming cognitive test and functional assessment scores against the site’s overall assessment and diagnostic conclusions. Per protocol, the external reviewers checked the first five participants for whom data collection and entry were completed at each site and subsequently every 5th participant, was randomly selected using Google’s Random Number Generator. The external reviewers have the option to place inquiries to the recruiting sites if the participant’s cognitive and/or functional instrument scores, captured in the Overall Assessment on the Consensus Form, do not seem consistent with the consensus diagnoses. External reviewers sign off on consensus diagnoses after the resolution of any inquiries.

In cases of borderline cognitive status—such as differentiating between subjective cognitive complaint (SCC) and mild cognitive impairment (MCI)—external reviewers may use this inquiry process to clarify the clinical rationale behind a site’s diagnosis. These clarifications are particularly valuable when diagnostic decisions rely on qualitative or contextual factors not captured in instrument scores. To support transparency in such cases, the revised Data Collection Packet (DCP v.2) now includes a dedicated free-text field for sites to record clinical observations relevant to diagnostic reasoning.

## Methods

At the time of data analysis, ACAD had one coordination site (University of Pennsylvania, single IRB #843791 covered all US recruitment sites), five US recruitment sites (Columbia University, University of Massachusetts Boston, University of Southern California and Southern California Eye Institute, University of California San Francisco, University of California San Diego), and two Canadian recruitment sites (University of British Columbia, REB #H21-00990; Centre of Addiction and Mental Health, REB #021/2021). Other supporting sites (e.g., non-recruitment sites) included Boston University, Brigham and Women’s Hospital, Icahn School of Medicine at Mount Sinai, Stanford University, and the University of Washington (National Alzheimer’s Coordinating Center). All participants have given consent to participate in the present study.

To check the consistency of the diagnostic process across all 7 ACAD sites, we had inter-rater reliability reviewers (B.L.T. and G.Y.R.H.) perform an additional review on cases that had already gone through the full consensus plus inter-rater reliability review process. We conducted an additional round of diagnostic logic review to develop a rating of inter-rater reliability, with the reliability tested with Cohen’s kappa.

At the time of this study, 88 participants from the ACAD study had undergone the site consensus process followed by approval by external reviewers. An ACAD Data Management Core member, independent of recruiting sites and blinded by study ID, created a subsample consisting of 6 participants from each of the 7 recruiting sites. These 6 represented cognitively healthy controls (which could include subjective cognitive complaint, SCC) and individuals with cognitive impairments, such as AD and MCI.

No site had fewer than 7 participants available for selection, allowing for the consistent sampling of 6 participants per site. From each site, the first five participants with completed REDCap data entry were selected, and one additional participant was randomly chosen from the next five enrolled, using Google’s random number generator. This approach ensured temporal distribution across recruitment waves—capturing participants enrolled early, mid-phase, and later in the study—to detect any systematic shifts in diagnostic classification over time. Overall, the sites had entered 561 consensus diagnoses into REDCap at the time of the inter-rater reliability review, however, only 550 had a complete data set from which this representative subset was drawn.

The high-level demographics (age and educational level) and cognitive and functional test scores for each participant were organized into a comprehensive spreadsheet for the inter-rater reliability reviewers. These data included the clinical dementia rating scale (CDR)^4^, modified mini-mental state examination (3MS)^5^ for Korean-speaking participants, or Cognitive Abilities Screening Instrument for all others (CASI-1—English and Vietnamese^6^; CASI-2—Cantonese, Mandarin^7–9^), clock drawing^10^, common objects memory test (COMT)^11^, geriatric depression scale (GDS)^12^, and functional assessment scale (FAS)^13^ scores, recapitulating the information available for external reviewers.

Due to variations in site logistics and pandemic-related restrictions, participant assessments were conducted using both in-person and remote methods. Specifically, all Vietnamese-speaking participants were evaluated remotely, while Korean-speaking participants were assessed in person. However, given that language group and assessment modality were confounded, and no subgroup had sufficient representation across both methods, we were unable to compare the diagnostic sensitivity or specificity between in-person and remote assessments.

The secondary reviewers independently designated agreement with the consensus diagnoses, blinded to the site identities. When they registered disagreement with a consensus diagnosis, they designated what diagnosis they would have chosen instead. This approach echoed the process used by the first round of external reviewers, to weigh the diagnosis toward the site’s consensus, where more details might be discussed than we select to review from the consensus form.

Cohen’s Kappa coefficient^14,15^ was used to evaluate the concordance between two raters while considering the potential occurrence of chance agreement. The measure estimates the level of agreement that goes beyond what would be anticipated by chance.

A kappa coefficient nearing 1 signifies a high level of agreement that is beyond what would be expected by chance, whereas values closer to 0 imply agreement that is no better than random. This measure is essential for guaranteeing consistency and reliability in review methods, which demonstrates the transparency of standards, the proficiency of reviewers, and the intricacy of the work being undertaken. Cohen’s kappa offers a standard approach to improve the reliability of research findings and systematic reviews by measuring the level of agreement among reviewers^15^.

For this study, Cohen’s Kappa measured the reliability of consensus diagnoses and overall assessments among recruiting sites as a proxy for accuracy and uniformity in the assessment process.

## Results

The 42 participants identified for the inter-rater reliability reviewers to assess consisted of 31% men and 69% women. Participants’ ages ranged from 60 to 92 years. The educational backgrounds of the participants were diverse, ranging from some high school to advanced doctoral degrees. A detailed breakdown of the demographic characteristics of the 42 participants is provided in Table 1. Table 2 further illustrates the cognitive test score ranges by language, illustrating how linguistic differences impact performance across the ACAD cohort.

**Table 1:** Characterization of the sample

Demographic variable	*n* (%)	Mean (standard deviation)
Age	42 (100%)	72.36 (8.33)
Sex at birth		
Men	13 (31.0%)	–
Women	29 (69.0%)	–
Education		
Some highschool	4 (9.53%)	–
High-school diploma	9 (21.43%)	–
Some college or technical school	10 (23.81%)	–
Undergraduate degree	15 (35.71%)	–
Graduate degree	4 (9.53%)	–
Language used in cognitive testing		Global cognition test score ranges (0–100)
English	6 (14.3%)	CASI-1 88–98.5
Korean	6 (14.3%)	3MS 48–95
Mandarin (simplified Chinese)	9 (21.43%)	CASI-2 52.7–99
Mandarin (traditional Chinese)	15 (35.71%)	CASI-2 59.8–100
Cantonese (traditional Chinese)	2 (4.76%)	CASI-2 88.9–90.4
Vietnamese	4 (9.53%)	CASI-1 82.9–95.4

The table outlines demographic details, linguistic information, and a range of global cognitive test scores for 42 participants whose consensus diagnoses were reviewed in the inter-rater reliability exercise.

**Table 2:** Cognitive test score ranges by language

Language	English	Vietnamese	Mandarin (simplified Chinese)	Mandarin (traditional Chinese)	Cantonese (traditional Chinese)	Korean
*N*	6	4	9	15	2	6
Global CDR (0–3)	0–0.5	0–0.5	0–2	0–1	0–0	0–2
CDR sum of boxes (0–18)	0–0.5	0–0.5	0–12	0–5	0–0	0–14
FAS (0–126)	0–2	0–2	0–30	0–9	0–2	0–21
Global cognition test score (0–100)	CASI-1 88–98.5	CASI-1 82.9–95.4	CASI-2 52.7–99	CASI-2 59.8–100	CASI-2 88.9–90.4	3MS 48–95
COMT trial 1 (0–10)	2–9	5–8	2–7	0–9	5–6	1–7
COMT trial 2 (0–10)	6–10	5–9	3–8	2–10	7–9	1–8
COMT trial 3 (0–10)	7–10	8–9	1–9	2–10	7–10	1–9
COMT delayed recall (0–10)	5–10	7–8	0–9	0–10	7–9	1–10
COMT delayed recognition (0–20)	20–20	20–20	18–20	13–20	20–20	18–20
Category fluency—vegetables	8–14	14–19	3–22	5–24	11–16	5–11
Category fluency—animals	11–15	7–16	3–17	2–18	11–12	4–11
Clock drawing (0–15)	14–15	5–14	6–15	9–15	13–15	0–15

This table displays cognitive test score ranges for participants tested in English, Vietnamese, Mandarin (simplified and traditional), Cantonese, and Korean. Measures include CDR, FAS fluency, global cognition, COMT trials, delayed recall/recognition, category fluency (vegetable and animal), and clock drawing test scores. The ranges reflect the performance variability across languages in the ACAD cohort.

**Fig. 1:**
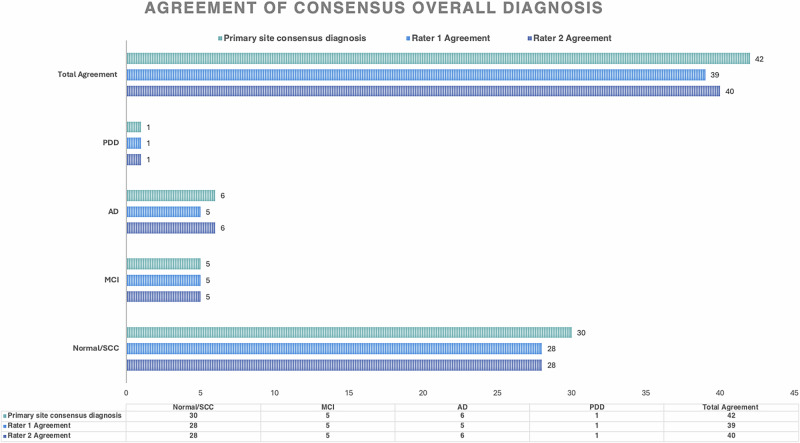
Inter-rater reliability reviewers’ agreement with the primary site consensus diagnosis. This figure displays the agreement on consensus diagnoses among the primary site and two raters across diagnostic categories (Normal/SCC, MC, AD, PDD). The *x*-axis counts the participants, and the *y*-axis lists the diagnoses. Blue bars show the primary site’s consensus, with darker and lighter shades indicating the agreement between Rater 1 and Rater 2. Total agreement is highlighted at the top, showing high consistency, especially in Normal/SCC and AD, with minor discrepancies in PDD. This aids in assessing the diagnostic alignment across different evaluators. SCC subjective cognitive complaint, MCI mild cognitive impairment, AD Alzheimer’s disease, PDD Parkinson’s disease dementia.

Cohen’s Kappa demonstrated that there was high reliability of consensus diagnoses and overall assessments among the recruiting sites. Agreement rates for consensus diagnosis and overall assessment stood at 88%, with a Cohen’s Kappa coefficient of 0.835 indicating Almost Perfect concurrence, per Landis and Koch (1977)^16^. This high level of agreement underscores the reliability of the diagnostic process across recruiting sites. Table 3 presents the details of the statistical analysis, including the agreement rate, Cohen’s Kappa coefficient, and standard error. Figure 1 provides detailed information regarding the degree of agreement among individual raters with the primary site consensus diagnosis. To ensure balanced site representation, six participants were selected from each of the seven ACAD recruiting sites for inter-rater reliability review. Table 4 summarizes the number of eligible participants per site and confirms that all sites had sufficient cases to support consistent sampling.

**Table 3:** Inter-rater reliability measures for diagnostic and overall assessments

	Observed agreement (%)	Cohen’s Kappa (lower & upper CI)	Standard error (SE)	Mispresented data
Diagnosis agreement	88	0.835 (0.700–0.971)	0.069	0.119
Overall assessment agreement	88	0.835 (0.700–0.971)	0.069	0.119

The table presents inter-rater reliability data for diagnosis and overall assessment agreements in a clinical study, using Cohen’s Kappa to quantify agreement levels. Both categories show an 88% observed agreement and a Cohen’s Kappa value of 0.835, indicating excellent reliability beyond chance. The standard error (0.069) and the 95% confidence interval, with a lower bound of 0.700 and an upper bound of 0.971, are also provided, indicating precise and reliable kappa estimations. Additionally, “misrepresented data” at 0.119 for both categories refers to inaccuracies in data representation, potentially due to errors in data entry or interpretation, affecting the study’s accuracy and reliability.

**Table 4:** Participants per site selected for inter-rater reliability review

Asian language sub-group	Consensus diagnoses completed	Diagnoses reviewed externally, “first round”	Diagnoses re-reviewed for inter-rater reliability
Chinese language or English-speaking	471	72	30
Vietnamese language or English-speaking	25	9	6
Korean language or English-speaking	54	7	6

This table summarizes the number of participants whose consensus diagnoses were completed across each Asian language subgroup and their subsequent inclusion in external reviews. The “first round” represents the initial external quality control step conducted by ACAD clinical core reviewers, while the “re-reviewed for inter-rater reliability” column reflects the final sample (*n* = 42) selected for the current inter-rater reliability analysis. Six participants were selected from each language subgroup to ensure balanced representation across the Chinese (Cantonese, Mandarin, English), Vietnamese (Vietnamese, English), and Korean (Korean, English) language tracks.

Among the 42 cases reviewed, diagnostic discrepancies arose in five. Three of these cases were initially designated as “Normal Control” by the sites, one involved a diagnosis of SCC and another of probable AD. Specifically, there was one instance of disagreement between probable AD and MCI, three instances of disagreement between Normal Control and MCI, and one instance of disagreement between SCC and MCI. Since SCC is considered a designation for otherwise Normal Control, the subsequent analysis concentrated on the four cases where disagreements were evident in diagnosing MCI.

The subsequent case descriptions elucidate the nature of these discrepancies and the rationale behind the final diagnostic decisions.

Case 1 (Probable AD vs. MCI): The participant was a 74-year-old male college graduate, assessed in Traditional Chinese without an informant. His Global CDR score was 0, with a Sum of Boxes score of 0.5, and his FAS score was 9. Initially, the site’s Overall Assessment indicated scores suggestive of dementia and functional impairment, and the participant was diagnosed with probable AD. The first external reviewers had assumed the site had other information to sway their diagnosis to dementia instead of MCI. Per the inter-rater reliability reviewers, abnormalities in the CDR and FAS scores did not seem severe enough to meet the criteria for dementia.

Case 2 (Normal Control vs. MCI): The participant was a 75-year-old female with technical school education, assessed in Simplified Chinese without an informant. Her Global CDR score was 0, and her Sum of Boxes score was 0, which initially led to an Overall Assessment that her cognition and functional abilities were intact. The site made a diagnosis of “Normal Control.” The first round of external reviewers approved the diagnosis. However, an inter-reliability reviewer identified that her COMT score, particularly with delayed recall of 7, was below normal limits.

Case 3 (Normal control vs. MCI): The participant is a 74-year-old female with technical school education, assessed in Simplified Chinese without an informant. Her Global CDR score was 0, the Sum of Boxes score was 0, and her scores on the CASI and other cognitive tests were above the dementia cutoffs. Initially, the Overall Assessment stated that her cognition and functional abilities were intact, and the site classified her as being a Normal Control, with the external reviewers approving this diagnosis, but subsequent inter-rater review noted borderline low scores on CASI, animal fluency, and COMT delayed recall that suggested that cognitive impairment might be present.

Case 4 (Normal control vs. MCI): The participant was a 76-year-old male college graduate assessed in Korean without an informant. His Global CDR score was 0, and his Sum of Boxes score was 0.5. The site’s overall assessment found that his cognition and functional abilities were intact, and he was classified as Normal Control, with approval by the external reviewers. However, an inter-rater reliability reviewer identified abnormalities in the CDR and FAS scores, indicating that the impairment exceeded normal limits.

Case 5 (SCC vs. MCI): The participant was a 73-year-old female college graduate assessed in Traditional Chinese, with a close friend serving as an informant. Her global CDR score was 0, and her CASI-2 scores and category fluency task results were slightly below the normative range. The site’s Overall Assessment indicated that her cognition and functional abilities were intact, although she reported SCC. Initially, she was classified as having SCC based on these findings, with approval by the external reviewers. However, one inter-rater reliability reviewer disagreed with the Overall Assessment and classification, citing the borderline CASI-2 scores and the informant’s reports of cognitive difficulties that seemed to go beyond mere subjectivity.

Each of the discrepancies above was discussed between site staff and the inter-rater reliability reviewers by videoconference, leading to the following changes in our process:

The site revised the diagnosis on Case 1 from probable AD to MCI after discussion.

Cases 2–4: The consensus diagnoses were revised from Normal Control to MCI. These changes indicated to the ACAD Clinical Core that the first round of external reviewers may need to inquire more frequently about borderline decision-making by the sites, as that first set of reviewers assumed that the sites had information beyond what was available for review to make their decisions. We now request more information on the participants’ function, especially in borderline cases, to help differentiate the transition between control to MCI, and MCI to AD.

Case 5: The consensus diagnosis was revised from SCC to MCI. To improve differentiation between SCC and MCI, the ACAD Data Collection Packet now includes a brief questionnaire to elicit SCC symptoms more consistently across sites.

The discussions generally led to a change in the way the Overall Assessment options are presented to data entry personnel in REDCap (ACAD’s electronic database capture platform): instead of merely listing Cognitive and Function impairment levels, there is also a reminder of the most frequent corresponding final diagnosis that corresponds to that selection, e.g., “Cognitive test scores suggest cognitive impairment/insufficient decline in functional abilities for dementia diagnosis (SCC or MCI)” or “Cognitive test scores dementia/insufficient functional impairment for dementia (possible dementia).”

## Discussion

The results of this study offer preliminary support for the reliability of the ACAD study. The high overall agreement rate of at minimum 88% and Cohen’s Kappa coefficient of 0.835 emphasize the internal consistency of the ACAD consensus process across independent reviewers, rather than its robustness for classifying participants across the broader clinical population. These findings suggest that standardized site procedures and shared diagnostic criteria can achieve a high level of diagnostic agreement, even in a diverse, multilingual cohort. However, the limited sample size prevents broad inferences about classification accuracy or generalizability.

It is important to note that Cohen’s Kappa is a key metric for assessing rater reliability, as it adjusts for chance agreement and provides a more accurate measure of rater concordance than simple percentage agreements. A Kappa value of 0.835, according to Landis and Koch’s (1977)^16^ guidelines, reflects almost perfect agreement, which is particularly impressive given the varied nature of the assessments.

The observed 88% agreement further bolsters confidence in the reliability of consensus diagnoses for distinguishing between Normal Controls and Dementia. This excellent degree of agreement underscores the effectiveness of the standardized procedures, even with impartial reviewers who were unaware of the site details and questions posed by the primary external reviewers.

### Improvements to ACAD process

The inter-rater reliability reviewers engaged in real-time discussions with site staff that had not been conducted by the first external reviewers. Given the absence of what turned out to be key narrative information obtained by the site clinicians not being captured in the REDcap database, the ACAD Clinical Core has reassessed what further information should be available to external reviewers who are not mandated to review all participant records. Generally, more qualitative information about testing conditions and notes about the evaluation beyond instrument scores has been implemented into the Consensus Worksheet.

To further evaluate the effectiveness of these procedural enhancements, the ACAD Clinical Core has planned a second inter-rater reliability exercise following the enrollment of the first 500 participants under the revised Data Collection Packet (DCP v.2), with corresponding changes to the formatting of REDCap (the data entry platform). This new phase of recruitment, supported by U19 funding, commenced in early 2025 and incorporates improved site training and structured guidance for handling borderline diagnostic cases. While the core diagnostic criteria remained unchanged, the instructions for interpreting test results were clarified to improve rating consistency across sites. Site staff received additional training to align with these procedural updates. The forthcoming reliability assessment will help determine whether these updates enhance diagnostic consistency and reduce classification discrepancies across participating sites.

In current practice, all consensus diagnoses entered into REDCap must be reviewed and approved by external raters before being finalized. When there is a discrepancy between test scores, functional assessments, and the assigned diagnosis, external reviewers initiate a query using REDCap’s audit trail and communication features. Site teams are expected to provide clarifications, and the external reviewers will withhold approval until all issues have been satisfactorily resolved. To support this process, DCP v.2 includes a dedicated free-text field where sites can document qualitative information, such as behavioral observations, informant narratives, or contextual factors, that may explain diagnostic decisions not fully captured by standardized scores.

A key strength of this study lies in its methodological rigor. The blinding of inter-rater reliability reviewers to the participants’ site details and the first external reviewer’s inquiries granted objectivity to the task, enhancing the credibility of the results. This method validated the extensive evaluation process, yielding strong indicators of consistency among raters.

However, there are limitations to consider. While the sample size is sufficient for assessments of rater reliability, our findings may not be fully applicable to routine clinical settings for Asians, particularly in diagnosing a broader range of dementias beyond Alzheimer’s disease. A substantial portion of these evaluations were performed in non-traditional clinical settings, such as through Zoom meetings and home visits, which could potentially lead to variations in the evaluation process. In particular, certain language subgroups were exclusively evaluated through either remote or in-person methods (e.g., all Vietnamese participants were evaluated remotely, while all Korean participants were assessed in person). As such, we were unable to perform a meaningful comparison between assessment modalities with regard to diagnostic accuracy. This confounding factor should be addressed in future protocol phases, as more data becomes available. Assessments in these various environments may need to be further investigated, especially after the COVID pandemic, remote assessments have been implemented in broad clinical and research settings.

Another limitation is that the ACAD protocol does not require participants without dementia to provide an informant to corroborate functional assessment. This may have led to some of the discrepancies for Cases 2–5, when participants came in initially as a “control” but were subsequently found to have some deficiency in testing, but there were no informants to corroborate any functional limitations that the participants may not have recognized themselves.

Finally, while Cohen’s Kappa coefficient serves as a measure of rater reliability, it does possess its own set of limitations. It operates under the premise that every disagreement is significant, which may not always be true in practice. Incorporating measures that consider the degree of disagreements, such as weighted Kappa or intraclass correlation coefficients for continuous data, could be beneficial for future research.

The investigators separated out Normal and SCC into two diagnostic categories because SCC indicates an increased risk for future decline^17,18^. When Normal and SCC were included in the same diagnostic category, there were fewer discrepancies in our study.

The outcomes of this research carry implications for research endeavors. The strong inter-rater reliability observed supports employing the ACAD consensus process as a benchmark for protocols in cognitive disorders. This standardization is critical to ensuring patients receive consistent diagnoses for genomic and risk factor analyses. Under current conditions, where ACAD is able to determine Asian-specific biomarker ranges for various forms of dementia, standardized diagnostic procedures are crucial. Biomarkers hold the potential to significantly revolutionize dementia research and diagnosis by providing objective metrics that can enhance clinical assessments.

The ACAD study demonstrated high agreement among inter-rater reliability reviewers across sites and in multiple languages in reaching consensus diagnoses and Overall Assessments, showcasing the effectiveness of reviews in maintaining diagnostic uniformity. These results validate the study’s approach but also reveal areas for improvement in future evaluations. The ACAD study will continue to establish a foundation for future research on cognitive disorders in Asian populations.

## Data Availability

The datasets used and analyzed in this study are available from the corresponding author upon reasonable request. Recruitment resources, including the data collection packet, are currently accessible to ACAD members and will be made available to the broader research community in the future, both by request and via our website at https://acadstudy.org.
